# Cafestol and kahweol: coffee diterpenes with trypanocidal activity and cardioprotective effects

**DOI:** 10.3389/fcimb.2026.1885889

**Published:** 2026-08-07

**Authors:** Juliana Magalhães Chaves Barbosa, Bruna Macedo Simões, Fábio Junior Moreira Novaes, Maria Alice Esteves da Silva, Patrícia Bernardino da Silva, Francisco Radler de Aquino Neto, Claudia Moraes de Rezende, Kelly Salomão, Tatiana Galvão de Melo

**Affiliations:** 1 Laboratório de Biologia Celular, Instituto Oswaldo Cruz, Fundação Oswaldo Cruz, Rio de Janeiro, RJ, Brazil; 2 Laboratório de Ultraestrutura Celular, Instituto Oswaldo Cruz, Fundação Oswaldo Cruz, Rio de Janeiro, RJ, Brazil; 3 Laboratório de Química Analítica - LAQUA, Departamento de Química, Universidade Federal de Viçosa, Viçosa, MG, Brazil; 4 Laboratório de Análise de Aromas, Instituto de Química, Universidade Federal do Rio de Janeiro, Rio de Janeiro, RJ, Brazil; 5 LADETEC, Instituto de Química, Universidade Federal do Rio de Janeiro, Rio de Janeiro RJ, Brazil

**Keywords:** cafestol and kahweol, Chagas disease, *Coffea arabica* L., diterpenes, *Trypanosoma cruzi*

## Abstract

**Introduction:**

Chagas disease (CD), caused by *Trypanosoma cruzi*, remains a major public health challenge in Latin America, affecting more than 7 million people worldwide and approximately 100 million individuals at risk of infection. Current chemotherapy relies exclusively on benznidazole and nifurtimox, which present significant limitations, such as prolonged treatment regimens, severe side effects, and limited efficacy during the chronic phase of the disease. These drawbacks highlight the urgent need for novel therapeutic alternatives. In this context, natural products, particularly diterpenes, have attracted considerable attention due to their broad spectrum of biological activities, including antioxidant, anti-inflammatory, and antimicrobial effects. Cafestol (**CF**) and kahweol (**KW**), *ent*-kaurane diterpenes found in *Coffea arabica* L., are recognized for their cardioprotective effects; however, their potential cardioprotective properties remain unexplored in the context of neglected diseases. This study aimed to evaluate the potential of **CF** and **KW** as trypanocidal agents and modulators of parasite-induced cardiac damage.

**Methods:**

To investigate the trypanocidal potential of **CF** and **KW**, both compounds were tested individually and in combination (1:1) against two *T. cruzi* strains representing distinct discrete typing units (DTUs), Y and Tulahuen. Their activity was evaluated against bloodstream trypomastigotes, the infective stage, and intracellular amastigotes, the replicative stage in the mammalian host. An in vitro infection model using cardiac cells as host cells was also employed, allowing the investigation of the ability of these diterpenes to mitigate parasite-induced cellular damage.

**Results:**

The results demonstrated that the combination of **CF** and **KW** enhanced trypanocidal activity against both *T. cruzi* strains tested. The **CF**+**KW** mixture showed significantly greater activity than the individual compounds against intracellular amastigotes of the Tulahuen strain (IC₅₀ = 9.6 µg/mL) and bloodstream trypomastigotes of the Y strain (IC₅₀ = 10.4 µg/mL). Against intracellular amastigotes of the Y strain, the combination remained active after 48 h of treatment (IC₅₀ = 13.3 µg/mL).

**Discussion:**

Moreover, the **CF** and **KW** combination demonstrated superior efficacy in preserving sarcomere integrity in infected cardiac cells compared with the individual compounds. Based on these findings and considering their pharmacological potential, **CF** and **KW** represent promising candidates for the development of new therapeutic strategies.

## Introduction

Chagas disease (CD), or American trypanosomiasis, affects over 7 million people, leading to 10,000 deaths per year; approximately 100 million people live in areas at risk of contracting the disease (Cucunubá et al., 2024; WHO, 2025). The pathology of CD classically presents an initial acute phase, usually characterized by mild and nonspecific symptoms. However, approximately 3-10% of patients may develop myocarditis (Marin-Neto et al., 2023). In this phase, the innate immune response plays an important role in the anti-*T. cruzi* defense, partially controlling the infection (Cristovão-Silva et al., 2021).

Following the acute phase, the disease progresses to the chronic phase, during which many individuals remain asymptomatic, and the indeterminate chronic phase is established, presenting positive serology due to a sustained specific immune response. After several decades, 20-30% of infected individuals progress to the symptomatic chronic phase, with different clinical manifestations: cardiac, digestive, or neurological (Higuchi et al., 2003; Rassi et al., 2012; Nunes et al., 2024). Especially in cardiac involvement, chronic Chagas cardiomyopathy is the most clinically significant manifestation of the chronic phase, characterized by ventricular dilation, cardiac fibrosis, severe arrhythmias, thromboembolic events, and heart failure, with myocardial infarction also being evidenced (Marin-Neto et al., 2023).

Treatment of CD is currently limited to two main drugs discovered over half a century ago: Nifurtimox (Nif) and Benznidazole (Bz) (Brazilian Ministry of Health, 2018; Marin-Neto et al., 2023). However, these drugs present several limitations, such as severe side effects that contribute to prolonged treatment regimens, high treatment discontinuation rate, and despite showing good efficacy when administered soon after infection, both drugs are ineffective during the late chronic phase of the disease (DNDi, 2023). Furthermore, treatment response may depend on factors such as the patient’s age and immune response, as well as the resistance or susceptibility profile of the *T. cruzi* strain involved (Soeiro et al., 2024). In view of this, there is a growing need to develop new therapeutic strategies that are safer and more effective, especially in the chronic phase of the disease. Promising approaches include reducing conventional dosages and treatment duration of currently used drugs, repositioning and combination of drugs already approved for human use (Salomão and Castro, 2017).

Natural products and compounds derived from them with antiparasitic properties have long been studied, taking advantage of their known medicinal potential (García-Huertas and Cardona-Castro, 2021). In recent years, natural products have gained increasing prominence in scientific research as a promising source of bioactive compounds for the development of new treatments against a wide range of diseases (Orosco et al., 2024; Shang et al., 2025). Substances belonging to classes such as alkaloids, flavonoids, and terpenoids have demonstrated relevant biological activities in several experimental models (Newman and Cragg, 2020), and these natural products may be strong candidates for CD therapy. Among the prominent natural products, diterpenes have attracted attention for their broad spectrum of biological activities. They are organic compounds that have 20 carbon atoms (C_20_) derived from the union of 4 isoprene units (C_5_H_8_), which form different cyclic skeleton types found in nature (Islam et al., 2020). They exhibit a range of biological functions, including antioxidant, anti-inflammatory, antimicrobial, and anticancer activities, which have been associated with important therapeutic benefits in humans (Islam et al., 2016). Studies on diterpenes have shown that they can modulate the expression of genes involved in inflammatory response and cellular metabolism, providing a molecular basis for the development of drugs of plant origin.

Coffee is one of the most widely consumed beverages worldwide; therefore, even minor individual health effects may have significant implications at the population level (Poole et al., 2017). More recently, attention has focused on its bioactive constituents with functional properties, including caffeine, chlorogenic acids, and the diterpenes Cafestol (**CF**) and Kahweol (**KW**) (Novaes et al., 2025). **CF** and **KW** are two *ent-*kaurane furan-type diterpene alcohols; they differ structurally only by the presence of a double bond between C1 and C2 in **KW**. Both are present in all coffee plant (*Coffea* spp.) tissue, with the highest concentrations found in *Coffea arabica* L. beans, the most commercially relevant species (Martins et al., 2024; Novaes et al., 2023). These compounds have been widely studied for their beneficial biological properties, particularly their ability to combat oxidative stress and reduce inflammation, factors associated with several chronic diseases, including cardiovascular diseases (Ren et al., 2019; Silva et al., 2023). However, their application to neglected diseases remains unexplored. The presence of these diterpenes in coffee and evidence that **CF** and **KW** are absorbed and become systemically available following coffee consumption have been recurrent topics in studies investigating the therapeutic potential of this widely consumed beverage (Amiri et al., 2024; Novaes et al., 2023; Silva et al., 2023). These findings, together with the broad range of biological activities previously reported for **CF** and **KW**, support their pharmacological potential. Therefore, we hypothesized that **CF** and **KW** may exhibit antiparasitic effects in addition to their cardioprotective properties, representing promising candidates for CD therapy as well as valuable scaffolds for the development of synthetic analogues.

## Materials and methods

### Chemicals and materials

**Figure 1:**
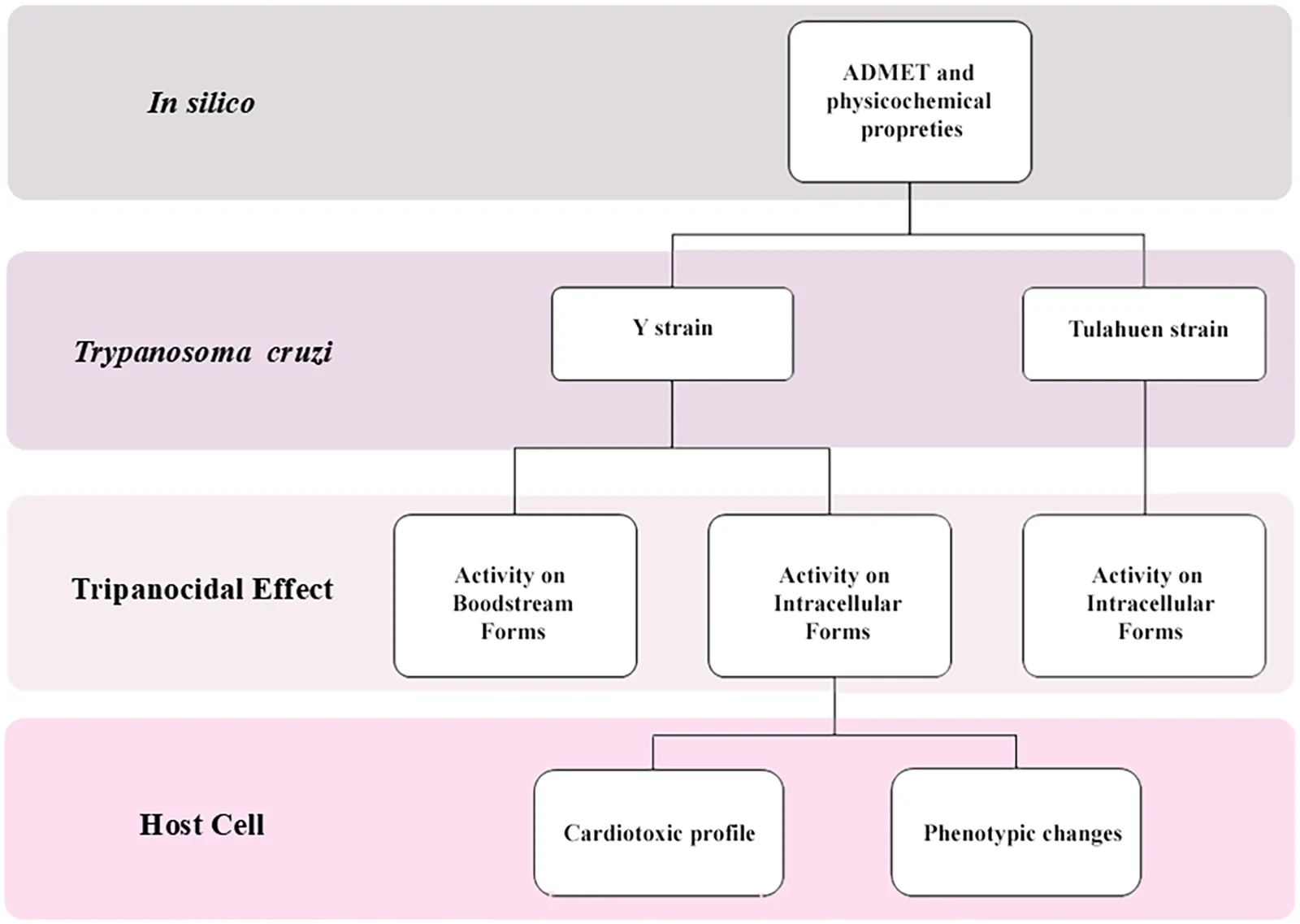
Methodological workflow employed in the present study: Evaluation of the potential use of coffee diterpenes as trypanocidal and cardioprotective agents.

**CF** and **KW** were isolated from green *Coffea arabica* beans as previously described (Novaes et al., 2020). An additional purification step for the **CF** and **KW** mixture (1:1) (**CF**+**KW**) was included by the recrystallization of these diterpenes in boiling acetone (prior to the evaluation **CF** and **KW** combination). The separation of **CF** from **KW** was achieved using recycling preparative high-performance liquid chromatography (HPLC). Finally, the isolated diterpenes were individually characterized by gas chromatography coupled to quadrupole mass spectrometry (GC-qMS), exact mass high-resolution mass spectrometry (HRMS), and ^1^H and ^13^C nuclear magnetic resonance (NMR) (Novaes et al., 2020) before their *in vitro* evaluation, as shown in **Figure 1**.

### Physicochemical properties, ADME parameters, and potential molecular targets prediction

**CF** and **KW** physicochemical properties and potential molecular targets prediction in *Mus musculus* were conducted, assessed by the SwissTargetPrediction web-based platform. Target prediction was performed using the platform default settings, generating a ranked list of putative targets based on chemical similarity to compounds with known bioactivity (Daina et al., 2019). The predicted targets were used for exploratory purposes, and those highlighted in the present study were selected according to their reported biological relevance in pathways associated with *T. cruzi* infection, inflammation, and cardiac remodeling. These predictions were used solely to generate mechanistic hypotheses and do not constitute experimental validation of target engagement. ADME parameters (Absorption, Distribution, Metabolism and Excretion) were also acquired using the same platform SwissADME (Daina et al., 2017).

### Activity against intracellular forms of *T. cruzi* (Tulahuen strain)

For the effect against intracellular forms (Tulahuen strain transfected with the *Escherichia coli* β-galactosidase gene), L929 cells were infected, using a 10:1 parasite/host cell ratio (Guedes-da-Silva et al., 2016), for 2 h, rinsed using saline to remove non-internalized parasites, and then incubated for 48 h at 37 °C, to establish the culture infection. Infected cultures were exposed to increasing non-toxic concentrations of **CF**, **KW**, and **CF**+**KW** mixture to determine the concentration that led to 50% lysis of the parasites after 96 h of incubation (IC_50_/96 h). Next, the absorbance was measured at 570 nm using a Spectra Max™ M3 spectrophotometer (Molecular Devices™, Sunnyvale, EUA). Results were expressed as the percentage of *T. cruzi* growth inhibition in compound-tested cells compared to untreated cells. In parallel, treatment with the reference drug (Bz) was always performed as a positive control (Freitas et al., 2020).

### Activity against bloodstream forms of *T. cruzi* (Y strain)

Bloodstream trypomastigotes of *T. cruzi*, Y strain, was obtained by cardiac puncture of infected Swiss Webster mice at the parasitemia peak, as previously described (Meirelles et al., 1986). The parasites were incubated at 37 °C in a 5% CO_2_ atmosphere with **CF**, **KW**, or **CF**+**KW** mixture (0.07 – 1000 µg/mL). After incubation for 24 h, parasite counts were performed in a Neubauer chamber by light microscopy and the activity of the compounds was expressed as the concentration that led to 50% lysis of the parasites after 24 h of incubation (IC_50_/24 h).

### Activity against intracellular forms of *T. cruzi* (Y strain)

Evaluation of the activity of **CF**, **KW**, and **CF**+**KW** mixture against intracellular forms was performed using primary cultures of heart muscle cells (HMCs) plated in 24-wells. Cultures were infected with bloodstream trypomastigotes (10:1 parasites/host cell ratio). After 24 h, the cultures were washed with phosphate buffer saline (PBS; Sigma-Aldrich™, St. Louis, MO, USA), once to remove non-adherent parasites and treated with **CF**, **KW**, and **CF**+**KW** mixture (12, 25, 50, and 100 µg/mL) for 24 h and 48 h. Finally, cultures were rinsed with saline, fixed in Bouin’s solution, stained with Giemsa stain, and the percentage of infection was quantified by randomly counting at least 200 cells per coverslip under light microscopy. The results were expressed by IC_50_ of the infection index, which corresponds to the multiplication of the percentage of infection by the number of parasites/infected cell.

### Cardiac cell model

HMCs were isolated from 18-day-old mouse embryos in a collagenase/trypsin solution and plated into 24-wells at a density of 1.5x10^5^ cells/well on glass coverslips coated with 0.01% gelatin (Sigma-Aldrich™). Cells were maintained in Dulbecco’s modified Eagle’s medium (DMEM) supplemented with 1 mM L-glutamine (Sigma-Aldrich™), 10% fetal bovine serum (FBS) (Cultilab, São Paulo, SP, Brazil), 2% chicken embryo extract and 2.5 mM CaCl_2_ at 37 °C in a 5% CO_2_ atmosphere. All procedures involving animals were approved by the Committee for the Use of Laboratory Animals of the Instituto Oswaldo Cruz (license LW-024/2023, Fundação Oswaldo Cruz).

### Mammalian cell cytotoxicity evaluation

Non-infected HMCs were incubated at 37 °C with **CF**, **KW**, and **CF**+**KW** mixture (25 – 100 µg/mL) for 24 h and 48 h. After treatment, PrestoBlue™ (Invitrogen™, USA) was added at a ratio of 1:10, the microplates were incubated for 2 h at 37 °C, and the fluorescence was measured at 540 and 590 nm, as recommended by the manufacturer, using a Spectra Max™ M3 spectrofluorometer (Molecular Devices™, Sunnyvale, CA, USA) for determination of CC_50,_ corresponding to the concentration that caused a 50% reduction in cell viability.

### Cytoskeletal analysis

*T. cruzi*-infected HMCs were treated for 48 h with the compounds at the concentrations corresponding to the IC_50_/48h of infection index in single treatment or in combination. Cells were fixed for 20 min at 4 °C with 4% paraformaldehyde (Sigma™) in PBS. Actin filaments (F-actin) were labeled with AlexaFluor 488-labeled phalloidin™ (Thermo Fisher Scientific™, Waltham, USA), and DNA was stained with 4′,6-diamidino-2-phenylindole dihydrochloride (DAPI; Sigma-Aldrich™). Slides were mounted and analyzed using a Zeiss Axio Imager M2 microscope™ equipped with the Apotome structured illumination system (Zeiss™, Oberkochen, Germany). For quantitative analysis, at least five random microscopic fields displaying comparable cell confluence were evaluated per experimental condition. Cardiomyocytes were classified as presenting either non-preserved sarcomeric structure or preserved/partially preserved sarcomeric structure based on the presence of recognizable F-actin organization and sarcomeric arrangement. The percentage of cells with preserved or partially preserved sarcomeric structures was determined for each experimental condition and used for comparative analyses among the groups.

### Statistical analysis

The obtained results are expressed as the mean ± standard deviation for each group from at least three independent experiments. The normality of the distribution of the variables was tested with the Shapiro–Wilk test. Between-group comparisons were made using one-way ANOVA followed by Tukey’s HSD *post-hoc* test or Kruskal-Wallis test followed by Dunn’s *post hoc* test (GraphPad InStat 8.0, GraphPad Software Inc. ™, La Jolla, USA). Values of p<0.05 were considered significant.

## Results

### Cafestol and kahweol exhibit favorable ADME properties, lack structural alerts, and display a lead-like profile

**Figure 2:**
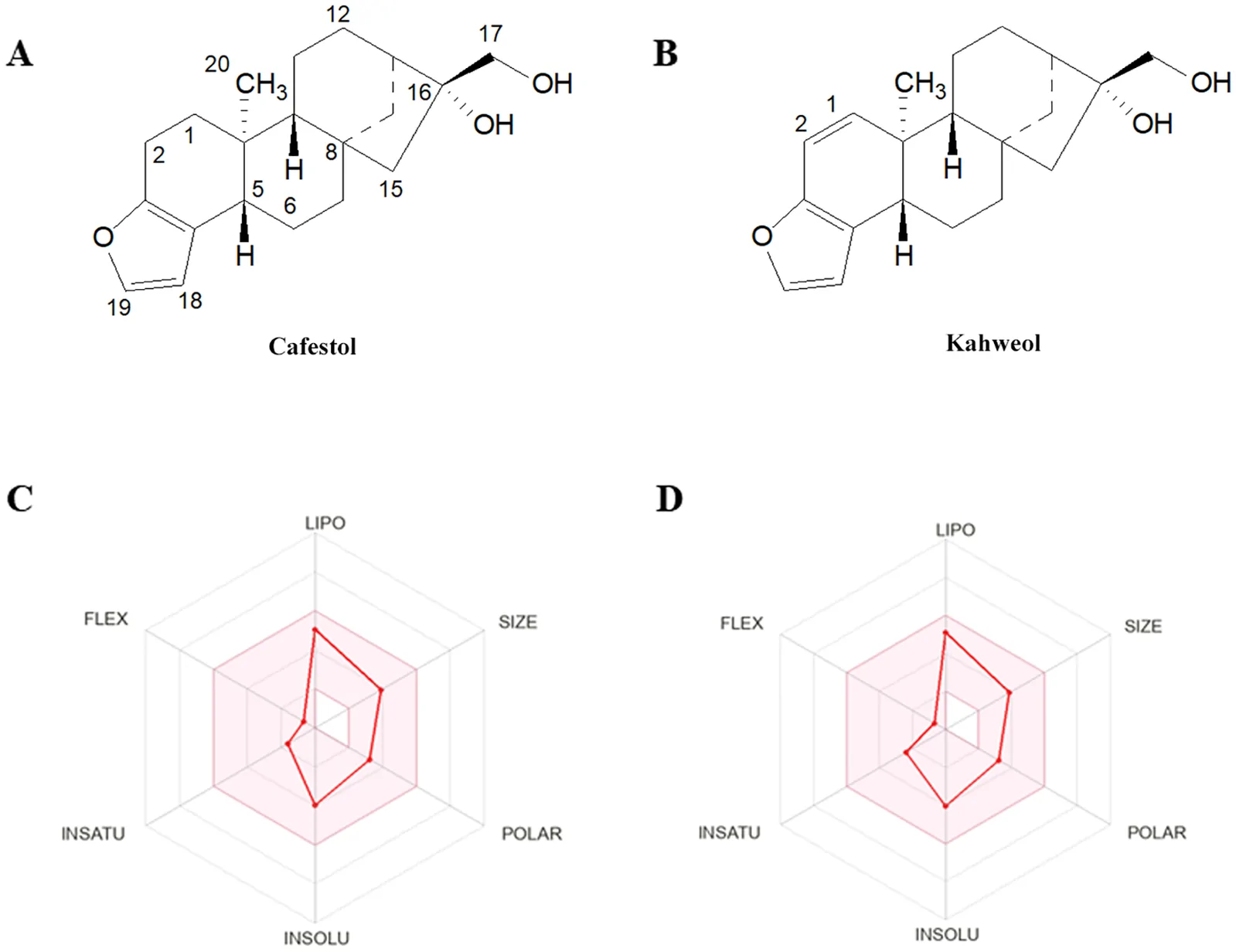
Chemical structure and bioavailability profile of arabica coffee diterpenes. **(A, B)** Chemical structures of **(A) CF** and **(B) KW**. **(C, D)** Bioavailability Radar plots for **(C) CF** and **(D) KW**, produced in SwissADME. The pink area represents the optimal range for each physicochemical property: lipophilicity (XLOGP3 between −0.7 and +5.0), size (molecular weight between 150 and 500 g/mol), polarity (tPSA between 20 and 130 Å^2^), solubility (log S ≤ -6), saturation (fraction of carbons in the sp3 hybridization ≥ 0.25) and flexibility (≤ 9 rotatable bonds) (Daina et al., 2017).

*In silico* predictions revealed that **CF** and **KW** comply with all Lipinski’s rules as they displayed a relatively low molecular weight (MW = 314 and 316 g/mol, respectively), an appropriate number of hydrogen-bond donors (HBDs < 5), hydrogen-bond acceptors (HBAs < 10), a low polar surface area (tPSA < 79 Å^2^) and a desired lipophilicity profile (cLogP = 3.25 and 3.19, respectively) (Lipinski et al., 2001) (**Figure 2**; **Table 1**). ADME predictions suggested that **CF** and **KW** are permeable to the blood-brain barrier (BBB) and display high intestinal absorption potential (**Table 1**). Regarding drug metabolism, neither compound was predicted to inhibit multiple CYP isoforms, although both showed potential inhibitory activity toward CYP2D6.

**Table 1:** Physicochemical and ADME properties of green *Coffea arabica* beans diterpenes.

Physicochemical properties		
	Cafestol	Kahweol
Formula	C20H28O3	C20H26O3
Molecular weight (g/mol)	316.43	314.42
Consensus Log P (o/w)	3.25	3.19
Log S (ESOL)	-3.96	-4.03
Solubility (mg/ml; mol/l)	3.44x10-2; 1.09x10-4	2.96x10-2; 9.41x10-5
Solubility class	Soluble	Moderately soluble
Pharmacokinetics		
GI absorption	High	High
BBB permeant	Yes	Yes
P-gp substrate	Yes	Yes
CYP1A2 inhibitor	No	No
CYP2C19 inhibitor	No	No
CYP2C9 inhibitor	No	No
CYP2D6 inhibitor	Yes	Yes
CYP3A4 inhibitor	No	No
Log Kp (skin permeation, cm/s)	-5.90	-5.80
Druglikeness		
Lipinski rule	Yes; 0 violation	Yes; 0 violation
Bioavailability score	0.55	0.55
Medicinal chemistry		
PAINS alerts	0	0
Brenk alerts	0	0
Leadlikeness	Yes	Yes
Synthetic accessibility	5.91	6.01

Besides, both compounds displayed favorable profiles in the SwissADME medicinal chemistry filters. Specifically, they showed no PAINS (Pan-Assay Interference Compounds) or Brenk alerts (Brenk Structural Alerts), indicating the absence of structural motifs commonly associated with assay interference, toxicity, or chemical instability. In addition, both were classified as *lead-like*, suggesting physicochemical characteristics compatible with further optimization during drug development. Regarding synthetic accessibility, the compounds presented SA scores of 5.9 and 6.0, respectively. This parameter ranges from 1 (very easy to synthesize) to 10 (very difficult) and provides a computational estimate of the relative synthetic complexity of a molecule. The observed values suggest moderate predicted synthetic accessibility, supporting the potential of these diterpenes as scaffolds for future medicinal chemistry optimization (Daina et al., 2017).

### Cafestol exhibits trypanocidal activity against both amastigotes and trypomastigotes of *T. cruzi*, while kahweol acts on trypomastigotes and enhances cafestol’s activity

Trypanocidal activity of **CF**, **KW**, and the **CF**+**KW** mixture were evaluated against trypomastigote and intracellular amastigote forms of two different strains *T. cruzi*. First, different concentrations of the compounds were tested against intracellular forms of *T. cruzi* (Tulahuen strain), and **KW** showed no detectable anti-*T. cruzi* activity against this strain, in these experimental conditions. Meanwhile, **CF** and **CF**+**KW** mixture exhibited effects, with IC_50_/96h values of 13.7 µg/mL and 9.6 µg/mL, respectively. The trypanocidal effect of **CF**+**KW** mixture was significantly higher than isolated **CF** (p= 0.0039).

**Figure 3:**
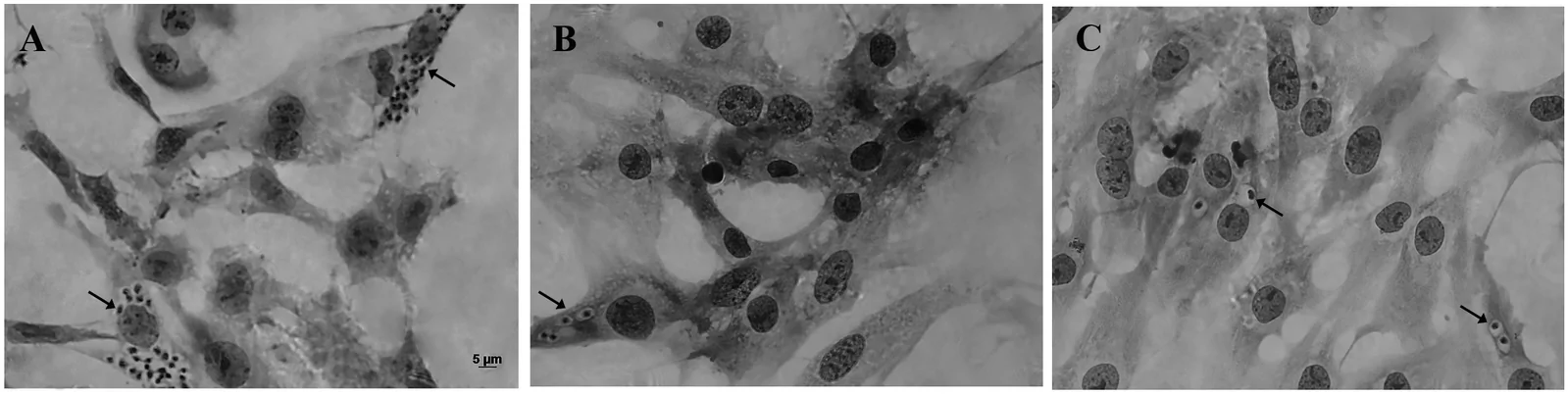
Effect of **CF**, **KW** on HMCs infected with *T. cruzi* (Y strain) after 72 h post infection (hpi) and 48 h of treatment. **(A)** Untreated *T. cruzi*-infected HMCs; **(B)** *T. cruzi*-infected HMCs treated with **CF** (IC_50_/48h: 16.5 μg/ml) and **(C)** *T. cruzi*-infected HMCs treated with **CF**+**KW** mixture (IC_50_/48h: 13.3 μg/ml). Black arrows indicate intracellular parasites.

After analyzing the activity of diterpenes against the blood trypomastigote form (Y strain), the results revealed that **CF** (IC_50_/24 h= 30.6 µg/mL), **KW** (IC_50_/24 h = 14.4 µg/mL), and a **CF**+**KW** mixture (IC_50_/24 h = 10.4 µg/mL) showed significant activity after 24 h of treatment, respectively. As observed before, the trypanocidal effect of **CF**+**KW** mixture was significantly higher than **CF** and **KW** alone (p ≤ 0.03). For the analysis of diterpenes activity against intracellular amastigotes of Y strain, primary cardiac cells were used as host cells. Quantification was performed after 24 and 48 h to determine the IC_50_ values for intracellular parasites. Under these experimental conditions, **KW** did not show trypanocidal activity at both treatment times (24 and 48 h). However, **CF** and **CF**+**KW** mixture showed biological activity on the amastigotes, in a time-dependent manner, since the **CF** and **CF**+**KW** mixture IC_50_ decreased from 36 to 16.5 µg/mL and from >50 to 13.3 µg/mL, respectively (IC_50_/24h *versus* IC_50_/48h). After 48 h of treatment, there was no significant difference between the trypanocidal effects of **CF** and **CF**+**KW** mixture (p > 0.05). The trypanocidal activity was expressed through the infection index that correlates the percentage of infection (number of infected cells) with the number of parasites per infected cell (**Figure 3**; **Table 2**).

**Table 2:** Activity of the diterpenes against bloodstream trypomastigotes of *T. cruzi* (Y strain) at 37 °C (IC_50_/24h) and activity against intracellular forms of *T. cruzi* after treatment for 24 and 48 h at 37 °C (IC_50_/24 h and IC_50_/48h, respectively).

Compounds	Bloodstream form	Intracellular form	
	IC50/24h (µg/mL)	IC50/24h (µg/mL)	IC50/48h (µg/mL)
Cafestol	30.6a ± 3.3b	36.0 ± 14.3	16.5 ± 2.9
Kahweol	14.4 ± 1.2	>50	>50
Cafestol + kahweol	10.4 ± 1.2*	>50	13.3 ± 3.4
Bz	2.30 ± 0.28^c^	0.48 ± 0.31d	2.60 ± 0.52^e^

^a^mean; ^b^standard deviation; ^c^Barbosa et al., 2021; ^d^Peres et al., 2023; ^e^Araujo-Lima et al., 2023 and * indicates statistical significance of **CF** and **KW** (*p* ≤ 0.03) determined by one-way ANOVA followed by Tukey’s HSD *post-hoc* test. The results obtained are from at least three independent experiments. Values of p < 0.05 were considered significant.

### Cafestol and kahweol show higher toxicity against *Trypanosoma cruzi* than against cardiomyocytes

**Figure 4:**
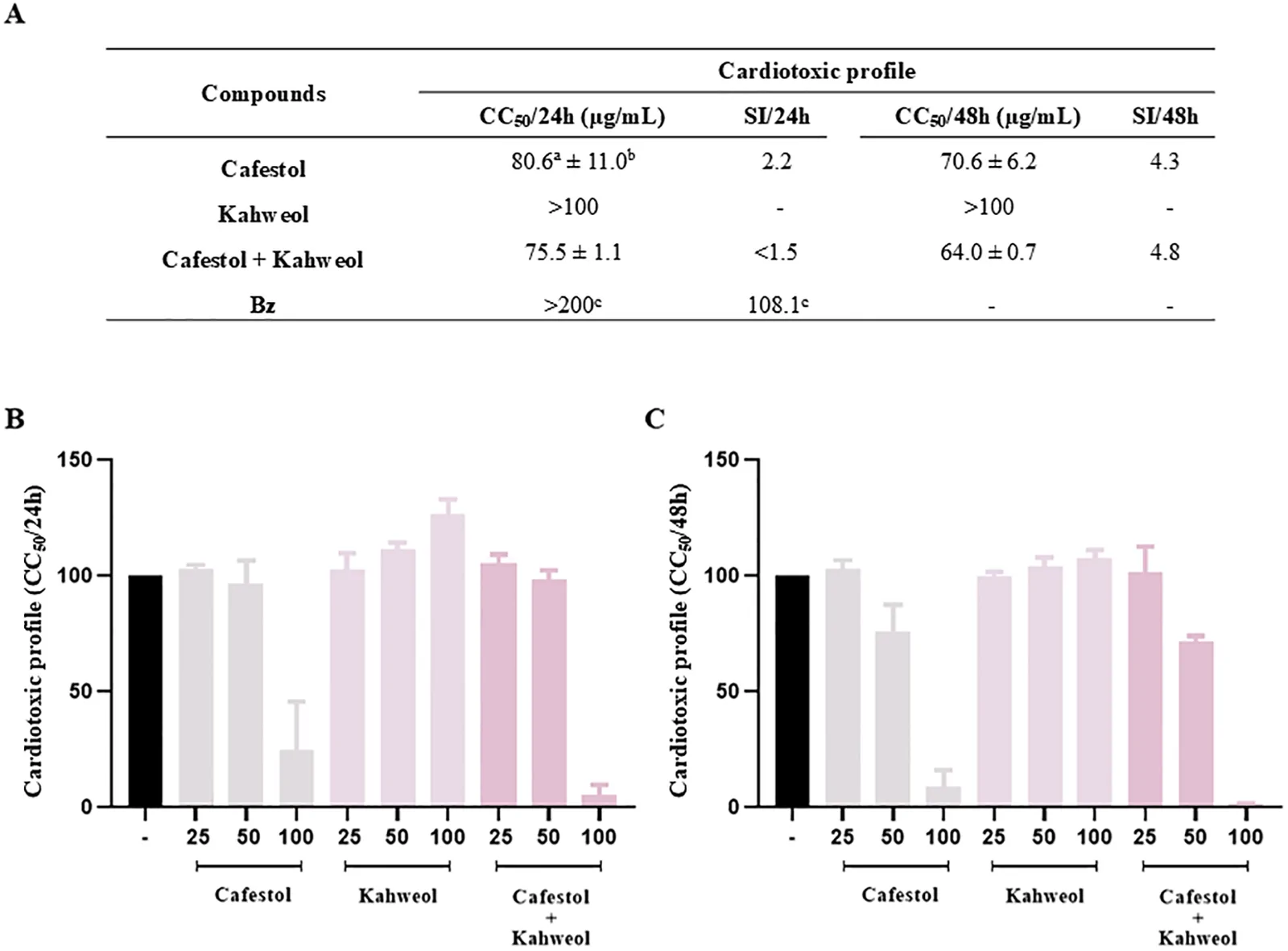
Cardiotoxic profile and **CF**, **KW**, and **CF**+**KW** mixture. **(A)** Table showing the cytotoxicity in HMCs (CC_50_) and selectivity Index (SI); Cardiotoxic profile of the compounds in **(B)** 24 h and **(C)** 48 h. Selectivity index (SI) = CC_50_ of uninfected HMCs/IC_50_ of intracellular parasites (Y strain). ^a^mean; ^b^standard deviation; ^c^Peres et al., 2023; The results obtained are from at least three independent experiments.

The toxic effect of the diterpenes were evaluated on HMCs, revealing that **KW** did not show cytotoxic effects on HMCs at either treatment time (24 and 48 h) (**Figure 4**). On the other hand, **CF** and **CF**+**KW** mixture induced a reduction in cell viability, particularly at the highest concentration tested (100 µg/mL) (**Figure 4**). Both **CF** and the **CF**+**KW** mixture were more toxic to the parasite than to the host cell, displaying selectivity index (SI/48 h) values of 4.3 and 4.8, respectively.

### The **CF**+**KW** mixture demonstrates superior cardioprotective effects compared to monotherapy, preserving sarcomeric organization in infected cardiomyocytes

**Figure 5:**
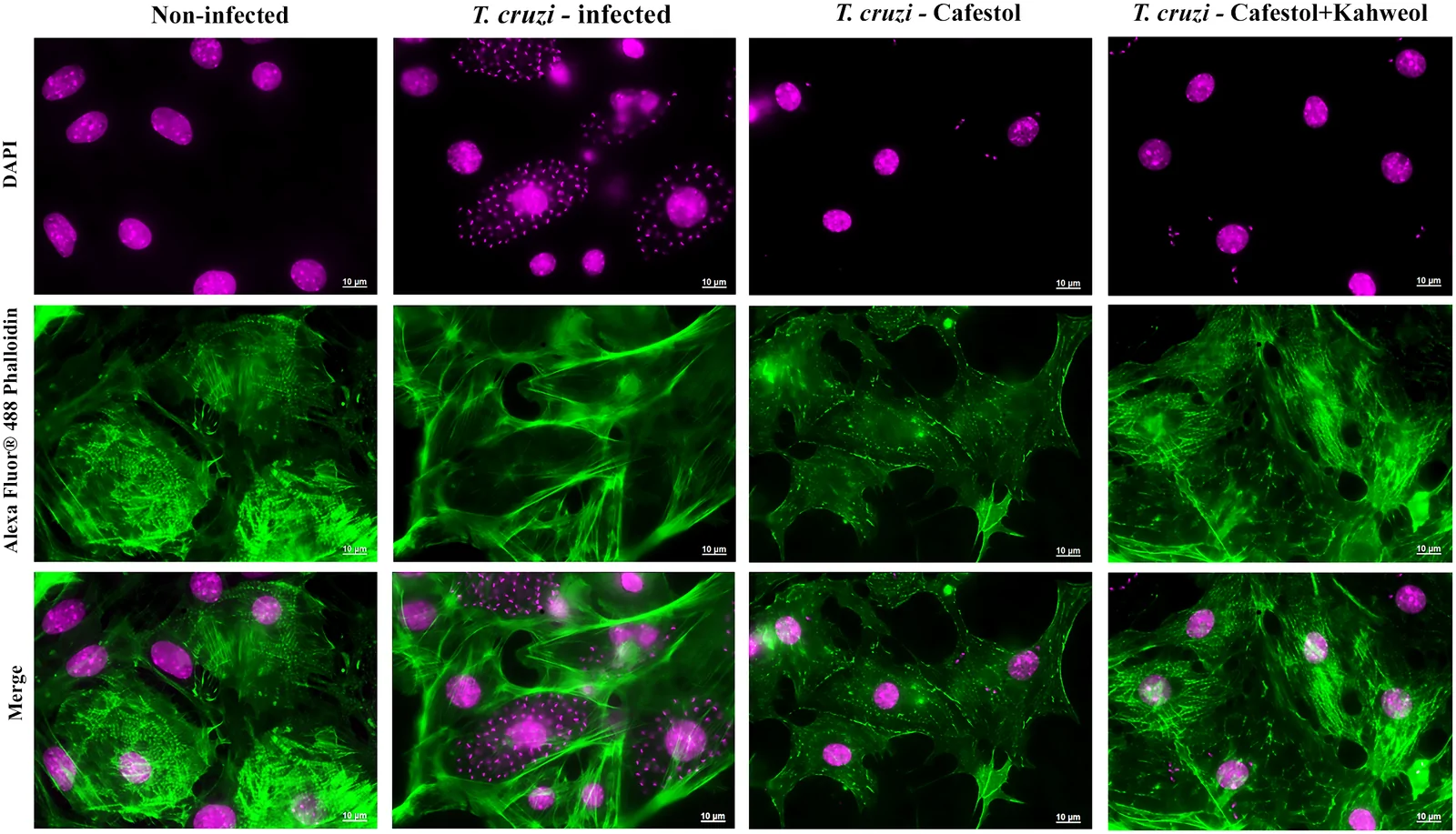
Cytoskeletal preservation in *T. cruzi*-infected HMCs treated for 48 h with **CF** (IC_50_/48h= 16.5 µg/mL) or the **CF**+**KW** mixture (IC_50_/48h= 13.3 µg/mL). Actin filaments and DNA were detected by phalloidin–FITC and DAPI, respectively. Of note, the rearrangement of actin structure in HMCs was also related to the reduction in intracellular parasite infection.

Our data revealed that uninfected control HMCs exhibited a typical distribution of well-defined striations upon phalloidin labeling of the F-actin cytoskeleton. After 72 h of infection with *T. cruzi*, a marked loss of these striations was observed in areas containing abundant intracellular amastigotes, consistent with previous reports describing cytoskeletal disorganization following *T. cruzi* infection (Pereira et al., 1993). Treatment with **CF** and the **CF**+**KW** mixture mitigated cytoskeletal damage in *T. cruzi*-infected HMCs. However, the **CF**+**KW** mixture appeared to be more effective than **CF** alone in preserving sarcomeric structures (**Figure 5**).

Quantitative analysis showed that the percentage of cells displaying preserved or partially preserved sarcomeric organization decreased from 74.0 ± 15.2% in uninfected controls to 0% in *T. cruzi-*infected untreated cultures (p < 0.0001). Notably, the **CF**+**KW** mixture was more effective than **CF** alone in maintaining sarcomeric integrity (**CF**+**KW**: 87.3 ± 7.6% *versus* **CF**: 64.8 ± 24.5%; p = 0.024) (**Supplementary Figure 1**).

### *In silico* prediction reveals targets related to inflammation, oxidative stress, and cytoskeletal remodeling, suggesting potential cardioprotective mechanisms of **CF** and **KW**

**Figure 6:**
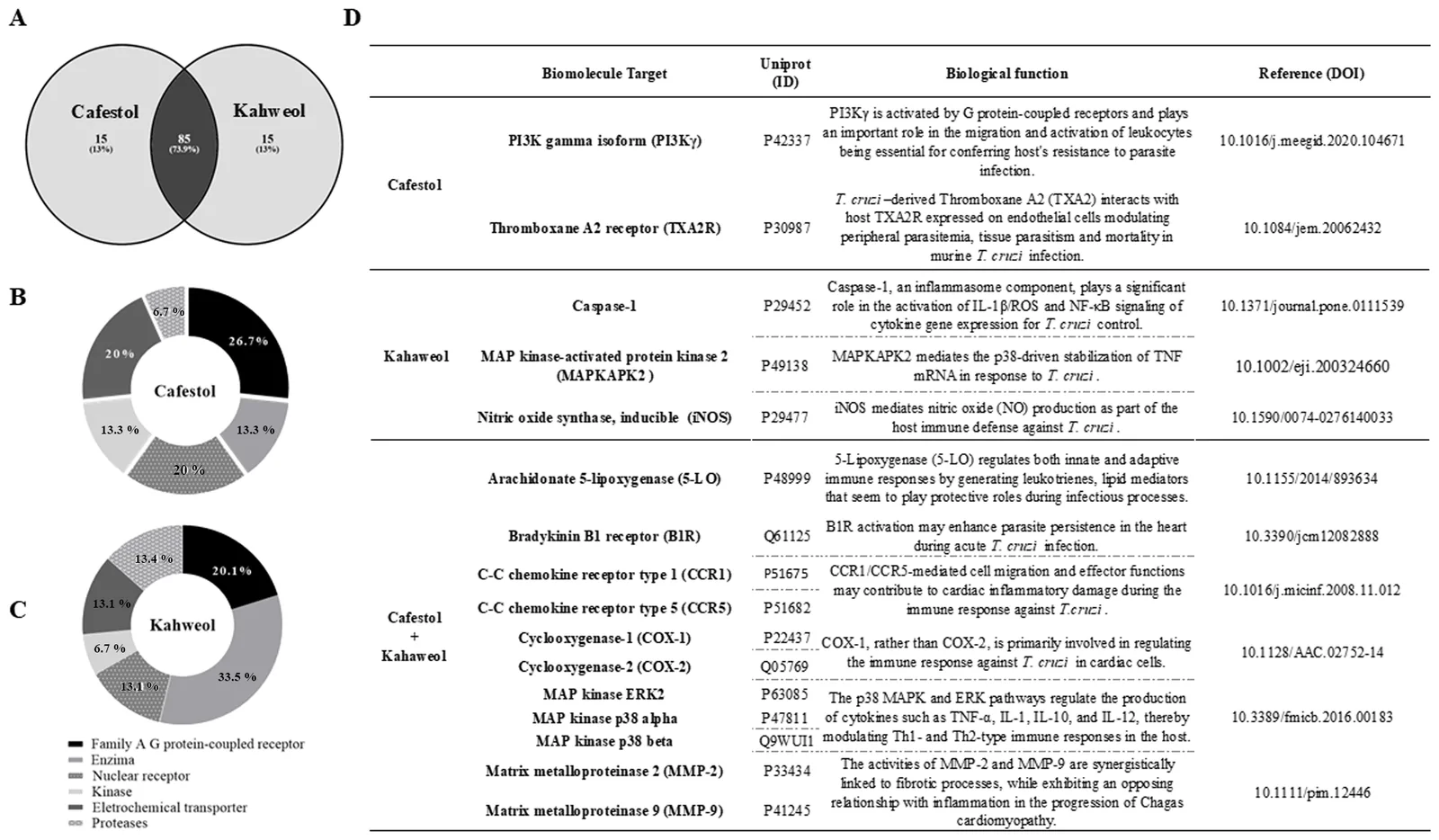
*In silico* analysis of predicted molecular targets of **CF** and **KW** in *Mus musculus*, conducted by SwissADME web-based platform. **(A)** Venn diagram showing common and uncommon targets of *Arabica coffee* diterpenes; Identification of biological class of target biomolecules of **(B) CF**, **(C) KW**, and **(D)** Table highlighting biomolecules previously described in the scientific literature as potential targets of **CF** and **KW**.

SwissTargetPrediction generated a list of up to 100 potential protein targets for **CF** and **KW** based on chemical similarity approaches. These predictions should be considered hypothetical and were used only to explore possible biological pathways that may be associated with the observed effects (**Supplementary Tables 1**, **2**, respectively). Most of these targets were shared between both compounds (73.9%), while 13% were exclusive to each compound. G protein-coupled receptors represented the most frequent predicted target class of both *Arabica coffee* diterpenes (**CF**: 26.7% and **KW**: 20%), followed by nuclear receptors (20%) and enzymes (33.5%) for **CF** and **KW**, respectively. Based on these results, the predicted targets were analyzed individually to identify potential molecular pathways of **CF** and **KW** that may contribute to the preservation of the cytoskeleton in *T. cruzi*-infected HMCs (**Figure 6**).

## Discussion

Chagas disease (CD) remains a significant public health challenge, predominantly affecting vulnerable populations in tropical and subtropical regions (Marin-Neto et al., 2023; WHO, 2025). Limited pharmaceutical investment, together with the biological complexity of the disease, has hampered progress toward both prophylactic vaccines and more effective therapeutic options (Soeiro et al., 2024). Currently, etiological treatment relies exclusively on the nitroheterocyclic drugs benznidazole (Bz) and nifurtimox (Nif), which are associated with considerable limitations, including frequent adverse events, prolonged treatment regimens, and low efficacy in the late chronic phase of infection. These limitations contribute to poor patient adherence and high treatment discontinuation rates (Bern, 2011; Salomão and Castro, 2017).

Considering these challenges, natural and semi-synthetic molecules have emerged as important sources of bioactive compounds with antimicrobial, antioxidant, and anticancer activities, often associated with fewer side effects and lower production costs (Pinazo et al., 2024). The microbicidal properties of several diterpenes isolated from natural products have been described, demonstrating antiviral, antibacterial, antifungal, and antiprotozoal activities, including activity against *T. cruzi*, thereby highlighting their therapeutic potential in infectious disease research (Alegre-Gómez et al., 2017; Islam et al., 2016).

In this context, **CF** and **KW** - diterpenes predominantly found in the tissues of the coffee plant (*Coffea* spp.) - have also been reported to exhibit a broad spectrum of bioactive properties, particularly antioxidant, anti-inflammatory, and anticarcinogenic effects (Ren et al., 2019; Silva et al., 2023; Novaes et al., 2025). In the present study, the trypanocidal activity of diterpenes derived from *Coffea* sp. was initially evaluated against the intracellular forms of *T. cruzi* of the Tulahuen strain (DTU VI), which is susceptible to the reference drug Bz (Romanha et al., 2010). For this reason, this strain is recommended as the first stage of screening assays for anti-*T. cruzi* bioactive compounds (Romanha et al., 2010; Soeiro et al., 2024). Subsequently, following our experimental workflow, we evaluated their activity against the bloodstream trypomastigote form of the *T. cruzi* Y strain (DTU II), which has been reported in the literature as partially resistant to Bz (Filardi and Brener, 1987). Evaluating compounds against *T. cruzi* strains from different DTUs is strongly recommended in the methodological pipeline for chemotherapy screening, as it broadens the understanding of their spectrum of activity (Vela et al., 2021).

In assays with intracellular forms of *T. cruzi* (Y and Tulahuen strains), only **CF** and the **CF**+**KW** mixture exhibited trypanocidal activity within the 10–20 µg/mL range, indicating consistent efficacy across these DTUs tested. In contrast, all tested compounds, including **KW**, were active against bloodstream trypomastigotes, suggesting that **KW** is more effective against the parasite’s extracellular form. Physicochemical analyses of *Coffea arabica* diterpenes indicated similar predicted solubility profiles for **CF** and **KW** according to SwissADME, with **CF** classified as soluble and **KW** as moderately soluble. In principle, differences in aqueous solubility may influence membrane permeability and, consequently, intracellular accumulation, which could in turn affect trypanocidal activity against intracellular parasite stages (Deprey et al., 2019). However, in the present context, this relationship remains speculative and cannot be inferred from the available data. Other factors, such as differences in cellular uptake, intracellular metabolism, subcellular distribution, or interactions with distinct molecular targets, may also contribute to the reduced efficacy of **KW** against intracellular forms of *T. cruzi* and warrant further investigation.

Since the trypanocidal activity was consistent across both DTUs, subsequent experiments were conducted using only the *T. cruzi* Y strain. This strain is widely employed in studies of host–cell interactions, particularly to investigate the effects of infection on the cytoskeleton of HMCs (Calvet et al., 2012). First, we evaluated the cytotoxicity profile of the compounds in uninfected HMCs. For both **CF** and the **CF**+**KW** mixture, the CC_50_ values were higher than the IC_50_ values determined for intracellular amastigotes, except for **KW**, which did not exhibit trypanocidal activity under the same experimental conditions. These findings indicate that trypanocidal activity was observed at concentrations that remained below those causing 50% host-cell death.

However, the selectivity indices (SI) obtained for **CF** and **KW** (approximately 4–5) are below those generally considered desirable for advanced stages of antiparasitic drug development. For earlier stages of hit identification, lower SI values (e.g., SI ≥ 10) may still be considered acceptable, depending on the biological context and therapeutic area (Soeiro et al., 2024). Nevertheless, the favorable medicinal chemistry profiles predicted by SwissADME - including the absence of PAINS and Brenk alerts, lead-like classification, and moderate synthetic accessibility - suggest that these molecules constitute promising scaffolds for future optimization. Such efforts may improve parasite selectivity, reduce host-cell toxicity, and enhance pharmacokinetic properties. Similar limitations have been reported for other diterpenes with anti-*T. cruzi* activity, which often require further structural refinement to achieve improved selectivity (Tabefam et al., 2018; Selener et al., 2024). Additionally, combination approaches with established trypanocidal drugs or other bioactive compounds may enhance antiparasitic efficacy while allowing dose reduction, thereby potentially improving the therapeutic window (Salomão and Castro, 2017).

Based on the results obtained, a 48-hour treatment with **CF** and the **CF**+**KW** mixture was selected for subsequent cytoskeletal analysis. During the intracellular development of *T. cruzi* in cardiomyocytes, disruption of the actin cytoskeleton and associated cell junction proteins has been consistently reported (Melo et al., 2004, 2006, 2019; de Melo et al., 2008; Pereira et al., 1993). Such structural alterations can impair electrical conduction and muscle contraction processes (Adesse et al., 2011; Calvet et al., 2012). The proper function of cardiac contraction–relaxation cycles and electrical signaling relies on the structural integrity of the myocardium, which is maintained by the organization of myofibrils and intercellular junctions (Marin-Neto et al., 2023).

Trypanocidal treatment of *T. cruzi*-infected human cardiomyocytes has been shown to counteract these infection-induced structural damages, exerting cardioprotective effects such as the restoration of myofibril structure and contractile function (Adesse et al., 2011; Silva et al., 2006; Barbosa et al., 2022). Thus, after 48 h of treatment, both **CF** and **CF**+**KW** mixture were able to mitigate the cytoskeletal damage caused by the parasite compared to infected untreated controls. Partially, this effect can be attributed to a reduction in intracellular parasitism. However, we observed that treatment with **CF**+**KW** mixture was more effective than **CF** alone in preserving myofibril organization, despite both conditions presenting similar infection rates (IC_50_/48 h). Based on these results, we hypothesize that the superior ability of the **CF**+**KW** mixture to preserve the actin cytoskeleton is not solely due to its trypanocidal effect.

To investigate the potential pleiotropic effects of these diterpenes, an *in silico* analysis was conducted to identify their possible molecular targets in *Mus musculus*. The predictions revealed that both **CF** and **KW** may interact with, and potentially modulate, key targets previously implicated in CD pathogenesis. Among these are matrix metalloproteinases (MMPs), which are involved in connective tissue remodeling and cardiac fibrosis (da Costa et al., 2019), as well as several important inflammatory mediators, including 5-lipoxygenase (5-LO), bradykinin receptor B1 (B1R), C-C chemokine receptors CCR1 and CCR5, cyclooxygenases COX-1 and COX-2, and mitogen-activated protein kinases (MAPKs) (Cristovão-Silva et al., 2021; MaChado et al., 2000).

Interestingly, **KW** alone was predicted to may interact with caspase-1 and inducible nitric oxide synthase (iNOS), both of which contribute to the immune response against *T. cruzi*, particularly through reactive oxygen species (ROS) production (MaChado et al., 2000). Thus, the potential downregulation of these two molecular targets by **KW** could contribute to reducing infection-induced oxidative stress, thereby mitigating heart cell damage (Paiva et al., 2018). Furthermore, the *in silico* analysis suggests that **KW** also targets mitogen-activated protein kinase-activated protein kinase 2 (MAPKAPK2), whose activation is crucial for the production of pro-inflammatory mediators such as tumor necrosis factor-alpha (TNFα), interleukin-1 beta (IL-1β), interleukin-8 (IL-8), interleukin-6 (IL-6), and interferon-gamma (IFNγ) (Kotlyarov et al., 1999). Additionally, MAPKAPK2 promotes actin polymerization and remodeling by mediating phosphorylation of heat shock protein 27 (Hsp27). In its unphosphorylated state, Hsp27 functions as a cap-binding protein for actin filaments, leading to inhibition of globular actin polymerization into filamentous actin (Soni et al., 2019). Thus, this biological function of MAPKAPK2 could directly contribute to the recovery of myofibrils damaged by infection, potentially restoring cytoskeletal integrity and contractile function in host cardiomyocytes (Soni et al., 2019).

In agreement with our *in silico* results, previous studies indicate that in macrophages, **KW** significantly reduces LPS-induced COX-2 expression and prostaglandin E2 production, primarily by inhibiting NF-κB activation through prevention of IκB degradation and suppression of IκB kinase activity, highlighting its anti-inflammatory potential (Kim et al., 2004). In addition, in neuronal SH-SY5Y cells, **KW** confers protection against 6-hydroxydopamine-induced oxidative stress by reducing ROS generation and caspase-3 activation. This neuroprotection effect is associated with upregulation of heme oxygenase-1 (HO-1) via activation of PI3K and p38 pathways, leading to Nrf2-dependent antioxidant responses (Hwang and Jeong, 2008). Together, these findings provide indirect support for the biological plausibility of the predicted targets and suggest that **KW** may exert anti-inflammatory and antioxidant effects. However, these mechanisms were not experimentally evaluated in the present study and should therefore be considered as hypotheses requiring further validation.

Despite the promising results, several limitations should be acknowledged. The lack of direct mechanistic analyses (e.g., ROS, cytokines, MMPs, MAPK pathways) prevents experimental confirmation of the proposed hypotheses in our experimental model. Although the **CF**+**KW** mixture exhibited greater cytoprotective activity than **CF** alone under the conditions tested, the nature of the interaction between these compounds (synergistic, additive, or antagonistic) cannot be determined from the present data, which would have strengthened the interpretation of the observed effects. Additionally, all biological experiments were performed *in vitro*. Although the cardiomyocyte infection model provides valuable mechanistic insights, the cardioprotective and therapeutic effects observed in the present study remain preliminary until validated *in vivo*. Future studies employing experimental models of CD will be necessary to determine whether these findings translate to reduced parasitemia, preservation of cardiac tissue architecture, and improvement of disease outcomes.

In addition to the biological effects observed *in vitro*, the *in silico* analyses provided further insights into the pharmacokinetic properties and drug-likeness profiles of **CF** and **KW**. ADME predictions suggested that both compounds possess high intestinal absorption potential, supporting their suitability for oral administration, and are capable of crossing the BBB (**Table 1**). This predicted BBB permeability is consistent with their low molecular weight, moderate lipophilicity, and low polar surface area. Nevertheless, the clinical relevance of this property for CD treatment remains unclear, given that chronic disease primarily affects the heart and digestive tract, while central nervous system involvement is generally limited to acute infection or disease reactivation in immunosuppressed individuals (Marin-Neto et al., 2023).

Therefore, our findings suggest that the trypanocidal activity against the intracellular form of the parasite is predominantly mediated by **CF** rather than **KW**. However, its combination with **KW** may also modulate key pathogenic mechanisms, including exacerbated inflammation and oxidative stress (Cristovão-Silva et al., 2021; Machado et al., 2000; Marin-Neto et al., 2023). Importantly, the presence of **KW** does not interfere with the trypanocidal effect of **CF**. Moreover, the **CF**+**KW** mixture was more effective in protecting cardiac cells from the deleterious effects of *T. cruzi* infection. Overall, these data enrich the repertoire of natural molecules with therapeutic potential against *T. cruzi* and introduce the **CF**+**KW** combination as an innovative strategy that combines antiparasitic activity with cardioprotection - an aspect particularly relevant for a disease whose main clinical outcome is chronic cardiomyopathy. Furthermore, this combination could potentially be used as an adjuvant to existing etiological treatments, such as Bz, to enhance therapeutic efficacy and reduce tissue damage in CD.

## Data Availability

The original contributions presented in the study are included in the article/**Supplementary Material**. Further inquiries can be directed to the corresponding author.

## Supplementary material

Supplementary Figure 1 Quantitative analysis of sarcomeric structure preservation in *T. cruzi*-infected HMCs after treatment with **CF** and the **CF**+**KW** mixture. HMCs were infected with *T. cruzi* and treated for 48 h with the compounds at concentrations corresponding to the IC_50_/48h of the infection index. Cells were classified according to sarcomeric organization as either non-preserved or preserved/partially preserved based on F-actin staining. Data represent the percentage of cells exhibiting preserved or partially preserved sarcomeric structures. At least five random microscopic fields with comparable cell confluence were analyzed per experimental condition. Results are expressed as mean ± SE. Statistical significance was determined by one-way ANOVA followed by Tukey’s HSD *post-hoc* test. Values of p < 0.05 were considered significant. ^****^p < 0.0001 *versus* NI (non-infected control); ^####^ p < 0.0001 *versus* Tc (*T. cruzi*-infected untreated control); ^&^ p < 0.05 for **CF**+**KW** mixture *versus* **CF**.
