# Supplementary material for: Cafestol and kahweol: coffee diterpenes with trypanocidal activity and cardioprotective effects
Source: Front Cell Infect Microbiol. 2026 Aug 7;16:1885889. doi: 10.3389/fcimb.2026.1885889 (PMC13493510; doi:10.3389/fcimb.2026.1885889)
Supplement: Supplementary file 1 [file DataSheet1.pdf]

SwissTargetPrediction

Target	Common name	Uniprot ID	ChEMBL ID	Target Class	Probability*	Known actives (3D/2D)
Estrogen receptor alpha (by homology)	Esr1	P19785	CHEMBL3065	Nuclear receptor	0.101613854776	33 / 0
Estrogen receptor beta (by homology)	Esr2	O08537	CHEMBL2995	Nuclear receptor	0.101613854776	37 / 0
11-beta-hydroxysteroid dehydrogenase 1	Hsd11b1	P50172	CHEMBL3910	Enzyme	0.101613854776	80 / 0
Mu opioid receptor (by homology)	Oprm1	P42866	CHEMBL2858	Family A G protein-coupled receptor	0.0	162 / 0
Kappa opioid receptor (by homology)	Oprk1	P33534	CHEMBL4329	Family A G protein-coupled receptor	0.0	150 / 0
Glucocorticoid receptor	Nr3c1	P06537	CHEMBL3144	Nuclear receptor	0.0	3 / 0
ADAM17 (by homology)	Adam17	Q9Z0F8	CHEMBL4379	Protease	0.0	5 / 0
G-protein coupled bile acid receptor 1 (by homology)	Gpbar1	Q80SS6	CHEMBL1255150	Family A G protein-coupled receptor	0.0	1 / 0
C-C chemokine receptor type 1 (by homology)	Ccr1	P51675	CHEMBL3872	Family A G protein-coupled receptor	0.0	37 / 0
Norepinephrine transporter (by homology)	Slc6a2	O55192	CHEMBL2370	Electrochemical transporter	0.0	113 / 0
Dopamine transporter (by homology)	Slc6a3	Q61327	CHEMBL2799	Electrochemical transporter	0.0	172 / 0
Serotonin transporter (by homology)	Slc6a4	Q60857	CHEMBL4642	Electrochemical transporter	0.0	184 / 0
Epidermal growth factor receptor erbB1 (by homology)	Egfr	Q01279	CHEMBL3608	Kinase	0.0	24 / 0
Glycogen synthase kinase-3 beta (by homology)	Gsk3b	Q9WV60	CHEMBL1075321	Kinase	0.0	27 / 0
Carbonic anhydrase XIII (by homology)	Ca13	Q9D6N1	CHEMBL2186	Enzyme	0.0	6 / 0
Carbonic anhydrase VII (by homology)	Ca7	Q9ERQ8	CHEMBL2216	Enzyme	0.0	5 / 0
cAMP and cAMP-inhibited cGMP 3',5'-cyclic phosphodiesterase 10A (by homology)	Pde10a	Q8CA95	CHEMBL1795126	Phosphodiesterase	0.0	24 / 0
Cyclooxygenase-1 (by homology)	Ptgs1	P22437	CHEMBL2649	Enzyme	0.0	6 / 0
Dual specificity mitogen-activated protein kinase kinase 1 (by homology)	Map2k1	P31938	CHEMBL5860	Enzyme	0.0	27 / 0
Orexin receptor type 2 (by homology)	Hcrtr2	P58308	CHEMBL2434818	Family A G protein-coupled receptor	0.0	1 / 0
Orexin receptor type 1 (by homology)	Hcrtr1	P58307	CHEMBL2434819	Family A G protein-coupled receptor	0.0	1 / 0
Liver glycogen phosphorylase (by homology)	Pygl	Q9ET01	CHEMBL3008	Enzyme	0.0	30 / 0

Target	Common name	Uniprot ID	ChEMBL ID	Target Class	Probability*	Known actives (3D/2D)
Arachidonate 5-lipoxygenase (by homology)	Alox5	P48999	CHEMBL5211	Oxidoreductase	0.0	12 / 0
Tyrosine-protein kinase JAK3 (by homology)	Jak3	Q62137	CHEMBL5250	Kinase	0.0	29 / 0
Delta opioid receptor (by homology)	Oprd1	P32300	CHEMBL3222	Family A G protein-coupled receptor	0.0	98 / 0
MAP kinase p38 alpha (by homology)	Mapk14	P47811	CHEMBL2336	Kinase	0.0	36 / 0
Cyclin-dependent kinase 1 (by homology)	Cdk1	P11440	CHEMBL4084	Kinase	0.0	15 / 0
HMG-CoA reductase (by homology)	Hmgcr	Q01237	CHEMBL2764	Oxidoreductase	0.0	63 / 0
Endothelin receptor ET-A (by homology)	Ednra	Q61614	CHEMBL2286	Family A G protein-coupled receptor	0.0	5 / 0
Aldo-keto reductase family 1 member C21 (by homology)	Akr1c21	Q91WR5	CHEMBL1075270	Enzyme	0.0	5 / 0
Macrophage migration inhibitory factor (by homology)	Mif	P34884	CHEMBL1926491	Enzyme	0.0	3 / 0
Beta-secretase 1 (by homology)	Bace1	P56818	CHEMBL4593	Protease	0.0	57 / 0
Butyrylcholinesterase (by homology)	Bche	Q03311	CHEMBL2528	Hydrolase	0.0	4 / 0
Serine/threonine-protein kinase Aurora-A (by homology)	Aurka	P97477	CHEMBL2211	Kinase	0.0	11 / 0
PI3-kinase p110-alpha subunit (by homology)	Pik3ca	P42337	CHEMBL2499	Enzyme	0.0	16 / 8
Cyclooxygenase-2 (by homology)	Ptgs2	Q05769	CHEMBL4321	Oxidoreductase	0.0	12 / 0
Tyrosine-protein kinase ABL (by homology)	Abl1	P00520	CHEMBL3099	Kinase	0.0	19 / 0
Neuronal acetylcholine receptor protein alpha-4 subunit (by homology)	Chrna4	O70174	CHEMBL4457	Ligand-gated ion channel	0.0	7 / 0
MAP kinase p38 beta (by homology)	Mapk11	Q9WUI1	CHEMBL4335	Kinase	0.0	9 / 0
Inosine-5'-monophosphate dehydrogenase 2 (by homology)	Impdh2	P24547	CHEMBL3169	Oxidoreductase	0.0	4 / 0
Sigma opioid receptor (by homology)	Sigmar1	O55242	CHEMBL3465	Membrane receptor	0.0	38 / 0
Adenosine deaminase (by homology)	Ada	P03958	CHEMBL3206	Hydrolase	0.0	14 / 0
Monoamine oxidase A (by homology)	Maoa	Q64133	CHEMBL3681	Enzyme	0.0	5 / 0
Carboxylesterase 2 (by homology)	Ces2c	Q91WG0	CHEMBL2217	Enzyme	0.0	3 / 0
Dipeptidyl peptidase IV (by homology)	Dpp4	P28843	CHEMBL3883	Protease	0.0	70 / 0

Target	Common name	Uniprot ID	ChEMBL ID	Target Class	Probability*	Known actives (3D/2D)
Dual-specificity tyrosine-phosphorylation regulated kinase 1A (by homology)	Dyrk1a	Q61214	CHEMBL4750	Kinase	0.0	6 / 0
C-C chemokine receptor type 5 (by homology)	Ccr5	P51682	CHEMBL3676	Family A G protein-coupled receptor	0.0	3 / 0
Histone deacetylase 6 (by homology)	Hdac6	Q9Z2V5	CHEMBL2878	Eraser	0.0	5 / 0
Phosphodiesterase 4B (by homology)	Pde4b	Q8VBU5	CHEMBL2272	Phosphodiesterase	0.0	4 / 0
High affinity cAMP-specific 3',5'-cyclic phosphodiesterase 7A (by homology)	Pde7a	P70453	CHEMBL2040702	Phosphodiesterase	0.0	7 / 0
Histone deacetylase 1 (by homology)	Hdac1	O09106	CHEMBL4001	Eraser	0.0	5 / 0
SGLT1 protein (by homology)	Slc5a1	Q9QXI6	CHEMBL1744523	Unclassified protein	0.0	9 / 0
Beta-2 adrenergic receptor (by homology)	Adrb2	P18762	CHEMBL3707	Family A G protein-coupled receptor	0.0	13 / 0
Beta-1 adrenergic receptor (by homology)	Adrb1	P34971	CHEMBL3440	Family A G protein-coupled receptor	0.0	6 / 0
Serotonin 1a (5-HT1a) receptor (by homology)	Htr1a	Q64264	CHEMBL3737	Family A G protein-coupled receptor	0.0	22 / 0
Beta-3 adrenergic receptor (by homology)	Adrb3	P25962	CHEMBL4030	Family A G protein-coupled receptor	0.0	4 / 0
Matrix metalloproteinase 9 (by homology)	Mmp9	P41245	CHEMBL2214	Protease	0.0	7 / 0
Matrix metalloproteinase-2 (by homology)	Mmp2	P33434	CHEMBL3095	Protease	0.0	7 / 0
Sodium channel protein type 9 subunit alpha (by homology)	Scn9a	Q62205	CHEMBL3414411	Voltage-gated ion channel	0.0	46 / 0
Nitric-oxide synthase, endothelial (by homology)	Nos3	P70313	CHEMBL2643	Enzyme	0.0	1 / 0
E3 ubiquitin-protein ligase Mdm2 (by homology)	Mdm2	P23804	CHEMBL3600279	Ligase	0.0	29 / 0
Androgen Receptor (by homology)	Ar	P19091	CHEMBL3056	Nuclear receptor	0.0	84 / 1
Dopamine D1 receptor (by homology)	Drd1	Q61616	CHEMBL3071	Family A G protein-coupled receptor	0.0	12 / 0
Metabotropic glutamate receptor 5 (by homology)	Grm5	Q3UVX5	CHEMBL1641352	Family C G protein-coupled receptor	0.0	5 / 0
Serine/threonine-protein kinase mTOR (by homology)	Mtor	Q9JLN9	CHEMBL1255165	Kinase	0.0	15 / 0
P2X purinoceptor 7 (by homology)	P2rx7	Q9Z1M0	CHEMBL5183	Ligand-gated ion channel	0.0	7 / 0
Smoothened homolog	Smo	P56726	CHEMBL6080	Frizzled family G	0.0	5 / 0

Target	Common name	Uniprot ID	ChEMBL ID	Target Class	Probability*	Known actives (3D/2D)
(by homology)				protein-coupled receptor		
Acetylcholinesterase	Ache	P21836	CHEMBL3198	Hydrolase	0.0	14 / 0
Mitogen-activated protein kinase 9 (by homology)	Mapk9	Q9WTU6	CHEMBL2034797	Kinase	0.0	9 / 0
Adenosine A2a receptor (by homology)	Adora2a	Q60613	CHEMBL2115	Family A G protein-coupled receptor	0.0	2 / 0
Anandamide amidohydrolase (by homology)	Faah	O08914	CHEMBL3455	Enzyme	0.0	4 / 0
Fatty acid synthase (by homology)	Fasn	P19096	CHEMBL1795189	Transferase	0.0	2 / 0
Neuronal acetylcholine receptor protein alpha-7 subunit (by homology)	Chrna7	P49582	CHEMBL3365	Ligand-gated ion channel	0.0	7 / 0
MAP kinase ERK2 (by homology)	Mapk1	P63085	CHEMBL2207	Kinase	0.0	19 / 0
DNA-dependent protein kinase catalytic subunit (by homology)	Prkdc	P97313	CHEMBL2176779	Kinase	0.0	5 / 0
11-beta-hydroxysteroid dehydrogenase 2 (by homology)	Hsd11b2	P51661	CHEMBL3490	Enzyme	0.0	1 / 0
Phosphatidylinositol 4,5-bisphosphate 3-kinase catalytic subunit gamma isoform (by homology)	Pik3cg	Q9JHG7	CHEMBL2189158	Enzyme	0.0	4 / 9
Alkaline phosphatase tissue-nonspecific (by homology)	Alpl	P09242	CHEMBL2660	Enzyme	0.0	3 / 0
Prostanoid EP1 receptor (by homology)	Ptger1	P35375	CHEMBL2181	Family A G protein-coupled receptor	0.0	3 / 0
Mast/stem cell growth factor receptor Kit (by homology)	Kit	P05532	CHEMBL2034798	Kinase	0.0	14 / 0
Thromboxane A2 receptor (by homology)	Tbxa2r	P30987	CHEMBL1795181	Family A G protein-coupled receptor	0.0	1 / 0
Tyrosine-protein kinase ZAP-70 (by homology)	Zap70	P43404	CHEMBL2034801	Kinase	0.0	2 / 0
Heat shock protein HSP 90-alpha (by homology)	Hsp90aa1	P07901	CHEMBL4197	Other cytosolic protein	0.0	4 / 0
Prostanoid EP4 receptor	Ptger4	P32240	CHEMBL2489	Family A G protein-coupled receptor	0.0	2 / 0
Prostanoid EP3 receptor	Ptger3	P30557	CHEMBL4336	Family A G protein-coupled receptor	0.0	2 / 0
Adenosylhomocysteinase (by homology)	Ahcy	P50247	CHEMBL2389	Enzyme	0.0	3 / 0
Muscarinic acetylcholine receptor M1 (by homology)	Chrm1	P12657	CHEMBL3733	Family A G protein-coupled receptor	0.0	14 / 0
Cathepsin K (by homology)	Ctsk	P55097	CHEMBL1075277	Protease	0.0	12 / 0
Macrophage colony-	Csf1r	P09581	CHEMBL5570	Kinase	0.0	8 / 0

Target	Common name	Uniprot ID	ChEMBL ID	Target Class	Probability*	Known actives (3D/2D)
stimulating factor 1 receptor (by homology)						
Sodium/glucose cotransporter 2 (by homology)	Slc5a2	Q923I7	CHEMBL1075302	Electrochemical transporter	0.0	6 / 0
Catechol O-methyltransferase (by homology)	Comt	O88587	CHEMBL3286068	Transferase	0.0	1 / 0
Muscarinic acetylcholine receptor M2	Chrm2	Q9ERZ4	CHEMBL3197	Family A G protein-coupled receptor	0.0	12 / 0
Vascular endothelial growth factor receptor 2	Kdr	P35918	CHEMBL3337	Kinase	0.0	3 / 0
ADP-ribosyl cyclase/ cyclic ADP-ribose hydrolase 1	Cd38	P56528	CHEMBL3425388	Enzyme	0.0	2 / 0
Serine/threonine-protein kinase 12 (by homology)	Aurkb	O70126	CHEMBL1075275	Kinase	0.0	7 / 0
Bradykinin B1 receptor (by homology)	Bdkrb1	Q61125	CHEMBL1250407	Family A G protein-coupled receptor	0.0	1 / 0
Histamine H3 receptor (by homology)	Hrh3	P58406	CHEMBL3263	Family A G protein-coupled receptor	0.0	17 / 0
Cannabinoid CB1 receptor (by homology)	Cnr1	P47746	CHEMBL3037	Family A G protein-coupled receptor	0.0	8 / 0
Cannabinoid CB2 receptor (by homology)	Cnr2	P47936	CHEMBL5373	Family A G protein-coupled receptor	0.0	8 / 0
Histamine H4 receptor (by homology)	Hrh4	Q91ZY2	CHEMBL5657	Family A G protein-coupled receptor	0.0	1 / 0
